# Long non-coding RNA FOXO1 inhibits lung cancer cell growth through down-regulating PI3K/AKT signaling pathway

**DOI:** 10.22038/ijbms.2019.31000.7480

**Published:** 2019-05

**Authors:** Xiaoqian Ding, Qiang Wang, Li Tong, Xin Si, Yong Sun

**Affiliations:** 1 Department of Respiratory, The Affiliated Hospital of Qingdao University, Qingdao 266000, Shandong, China; 2 Department of Radiology, Qingdao West Coast New Area Central Hospital, Qingdao 266555, Shandong, China

**Keywords:** Apoptosis, Cell proliferation, FOXO1, Metastasis, PI3K/AKT

## Abstract

**Objective(s):**

Lung cancer is one of the most common malignant tumors, which seriously threatens the health and life of the people. Recently, a novel long non-coding RNA (lncRNA) termed lncFOXO1 was found and investigated in breast cancer. However, the effect of lncFOXO1 on lung cancer is still ambiguous. The current study aimed to uncover the functions of lncFOXO1 in lung cancer cell proliferation, metastasis and apoptosis.

**Materials and Methods:**

LncFOXO1 expression levels in lung cancer tissues or cells were detected using qRT-PCR. Then, overexpression and knockdown vectors of lncFOXO1 were transfected into A549 cells to investigate the effect of lncFOXO1 on cell proliferation, invasion, migration and apoptosis. These experiments were assessed using MTT, colony formation, transwell, flow cytometry and western blot assays, respectively. In vivo experiment was performed to examine the tumor weight using Xenograft tumor model assay. The important pathway of PI3K/AKT was finally examined using western blot.

**Results:**

The decreased expression level of lncFOXO1 was observed in lung cancer tissues and cells (A549, H460, HCC827 and H1299). Knockdown of lncFOXO1 significantly promoted A549 cells viability, colony formation and invasion. However, lncFOXO1 overexpression obviously reversed the results. Moreover, lncFOXO1 overexpression induced A549 cells apoptosis by regulating Bax, cleaved-caspase-3 and Bcl-2. *In vivo* experiment revealed that lncFOXO1 overexpression inhibited tumor weight. Furthermore, lncFOXO1 knockdown promoted colony formation and mediated Myc and Cyclin D1 expressions by regulating PI3K/AKT signaling pathway.

**Conclusion:**

LncFOXO1 inhibited lung cancer cell proliferation, metastasis, and induced apoptosis through down-regulating PI3K/AKT pathway.

## Introduction

Lung cancer is one of the most common and serious malignant visceral tumors, which has become the chief cause of cancer death worldwide (1). To date, the cause of lung cancer is not yet fully understood, but mounting evidences demonstrate that long-term and heavy smoking are associated with the occurrence of lung cancer (2, 3). The manifestations of lung cancer are complex, and common symptoms often include coughing, hemoptysis, shortness of breath, and chest pains (4, 5). Due to the lack of effective molecular markers for early clinical diagnosis and treatment, the five-year survival rate of lung cancer is still very poor (6). Therefore, it is necessary to find a new molecular marker for the diagnosis and treatment of early lung cancer.

Long non-coding RNAs (lncRNAs) are defined as RNA molecule with a length of 200-100000 nucleotides, which does not encode proteins (7). Many studies have displayed that lncRNAs have an expression characteristic in various cancers, which are associated with tumor cells growth and metastasis (8-10). The results of a study showed that lncRNA H19 acted as an oncogenic gene in lung cancer, and lncRNA H19 overexpression was found to be associated with the poor survival in lung cancer patients (11). LncRNA that is encoded by maternally expressed gene 3 (MEG3) has been shown to inhibit lung tumorigenesis by controlling cell proliferation (12). Furthermore, Gao *et al.* demonstrated that lncRNA tissue factor pathway inhibitor 2 antisense RNA 1 (TFPI2AS1) suppressed lung cancer cell proliferation and migration (13). All these studies indicated the irreplaceable role of lncRNAs in lung cancer. LncFOXO1 is a novel lncRNA, which has been recently reported to inhibit the growth of breast cancer cells by regulating BRCA1-associated protein 1 (BAP1) (14). However, the effect of lncFOXO1 on lung cancer has not yet been fully reported.

In the present study, we have a strong interest in exploring the effect of lncFOXO1 on lung cancer. We examined the expression level of lncFOXO1 in lung cancer tissues and lung cancer cells by using qRT-PCR. Then, sh-FOXO1 and pc-FOXO1 vectors were transfected into A549 cells, and the biological functions of lncFOXO1 were investigated. *In vivo* experiment was performed to further explore the effect of lncFOXO1 on tumor weight using Xenograft tumor model assay. Finally, phosphoinositide 3-kinase (PI3K)/protein kinase B (AKT) signaling pathway was examined by using western blot. These findings will open a new field for early molecular diagnosis, prognosis and therapy of lung cancer.

## Materials and Methods

***Tissue samples***

The lung cancer tissue samples and corresponding adjacent tissue samples were obtained from twenty patients with lung cancer from The Affiliated Hospital of Qingdao University. The samples were collected from lung cancer patients (12 male and 8 female, aged from 39-70 years) from February 2016 to October 2016 who did not undergo preoperative radiotherapy or chemotherapy. These samples were collected and immediately preserved in liquid nitrogen for subsequent experiment. The study was approved by the local ethics committee of The Affiliated Hospital of Qingdao University, and all lung cancer patients who participated in this study signed the informed consent.

**Figure 1:**
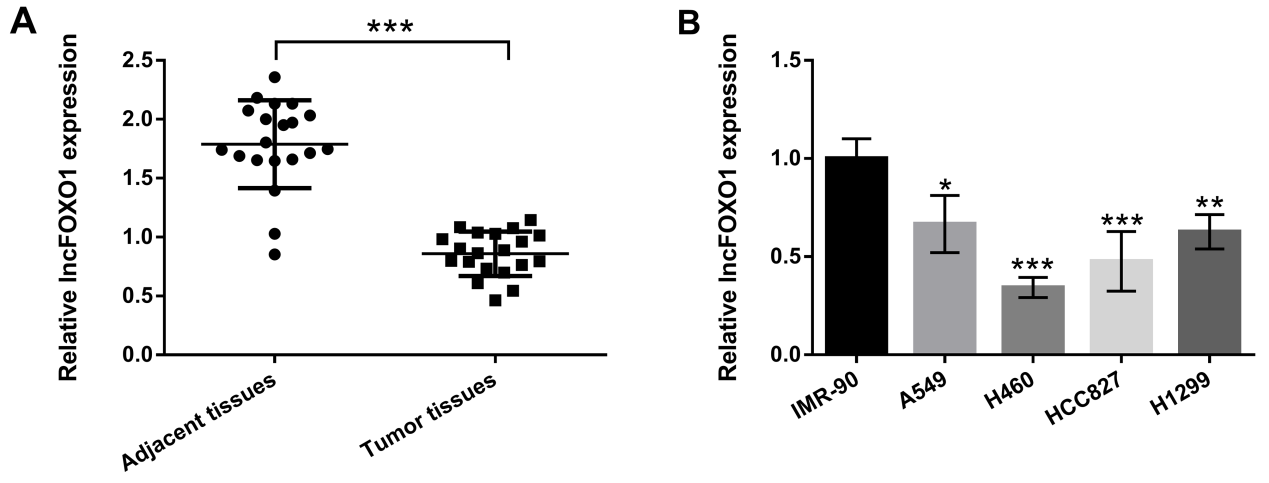
LncFOXO1 was down-regulated in lung cancer tumor tissues and cells. (A) The expression levels of lncFOXO1 in lung cancer tissues and adjacent tissues were examined by qRT-PCR; (B) The expression level of lncFOXO1 in lung cancer cells (A549, H460, HCC827 and H1299) and lung fibroblast IMR-90 cells were detected by qRT-PCR. Data are presented as the mean ± SD of three independent experiments; * *P*<0.05, ** *P*<0.01, *** *P*<0.001. LncFOXO1: long non-coding RNA FOXO1; qRT-PCR: quantitative real time PCR

**Figure 2:**
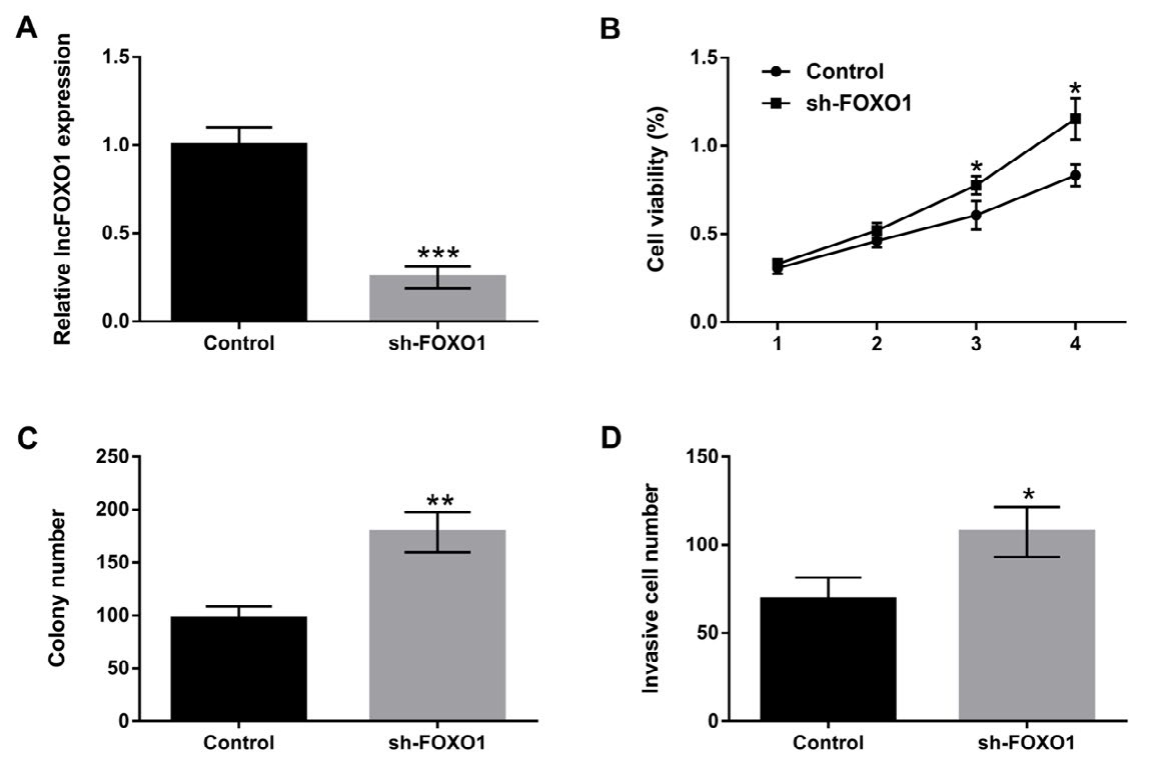
Knockdown of lncFOXO1 promoted cell proliferation and invasion. A549 cells were transfected with sh-FOXO1 vector. (A) The expression level of lncFOXO1 in control and sh-FOXO1 transfected cells was examined by qRT-PCR; (B) The cell viability in control and sh-FOXO1 transfected cells was detected by MTT assay; (C) The colony number in control and sh-FOXO1 transfected cells was detected by colony formation assay; (D) The invasiveness of A549 cells in control and sh-FOXO1 transfected cells was determined by Transwell assay. Data are presented as the mean ± SD of three independent experiments; * *P*<0.05, ** *P*<0.01, *** *P*<0.001

LncFOXO1: long non-coding RNA FOXO1; shRNA: short hairpin RNA; qRT-PCR: quantitative real time PCR; MTT: 3-(4, 5-dimethylthiazol-2-yl)-2 5-diphenyl-2Htetrazolium bromide

**Figure 3:**
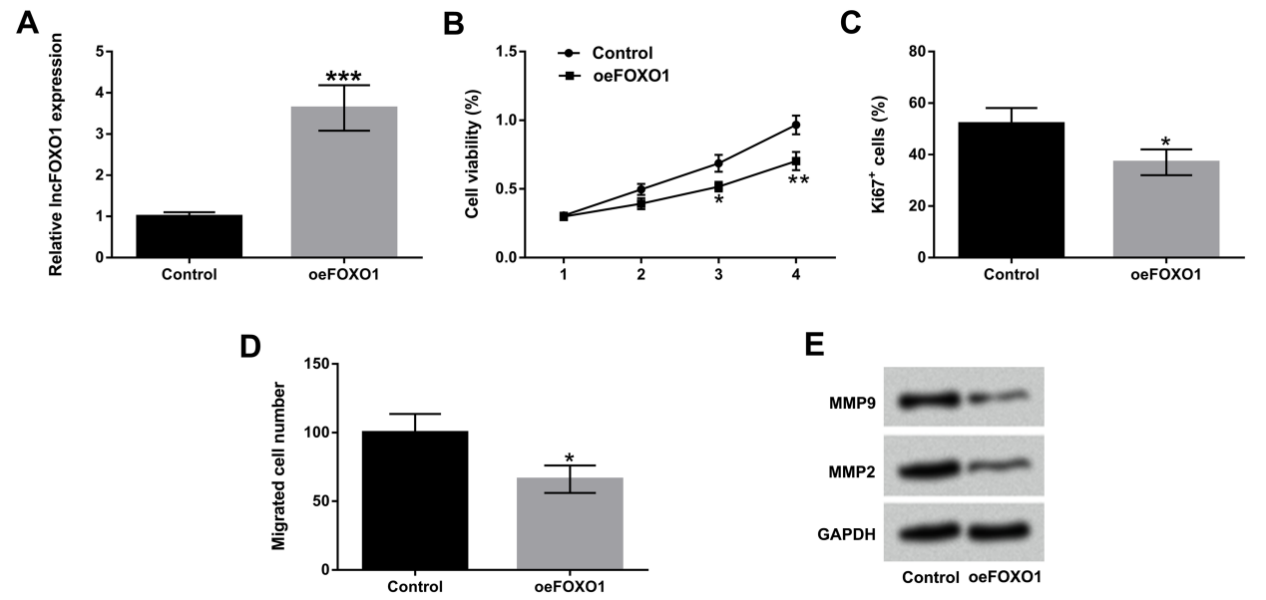
Overexpression of lncFOXO1 suppressed cell proliferation and migration. A549 cells were transfected with pc-FOXO1 vector. (A) The expression level of lncFOXO1 in control and pc-FOXO1 transfected cells was examined by qRT-PCR; (B) The cell viability in control and pc-FOXO1 transfected cells was detected by MTT assay; (C) The percentage of Ki67^+^ cells in control and pc-FOXO1 transfected cells was detected by flow cytometry assay; (D) The migration of A549 cells in control and pc-FOXO1 transfected cells was determined by Transwell assay; (E) The protein levels of MMP9 and MMP2 in control and pc-FOXO1 transfected cells were assessed by western blot. Data are presented as the mean ± SD of three independent experiments. * *P*<0.05, ** *P*<0.01, *** *P*<0.001

LncFOXO1: long non-coding RNA FOXO1; shRNA: short hairpin RNA; qRT-PCR: quantitative real time PCR; MTT: 3-(4, 5-dimethylthiazol-2-yl)-2 5-diphenyl-2Htetrazolium bromide; MMP: matrix metallopeptidase

**Figure 4:**
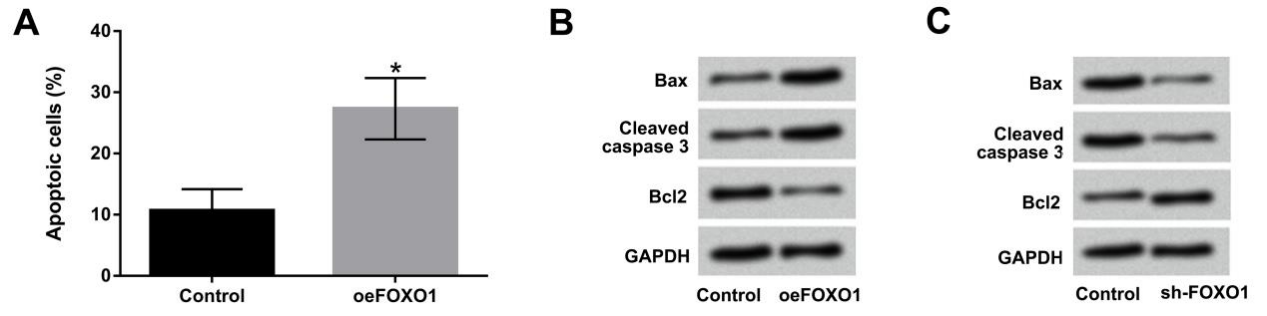
Overexpression of lncFOXO1 induced cell apoptosis in A549 cells. A549 cells were transfected with pc-FOXO1 and sh-FOXO1 vectors. (A) Cell apoptosis in control and pc-FOXO1 transfected cells was detected by flow cytometry; the protein levels of Bax, cleaved -caspase-3 and Bcl-2 (B) in control and pc-FOXO1 transfected cells and (C) in control and sh-FOXO1 transfected cells were analyzed by western blot assay. Data are presented as the mean ± SD of three independent experiments; * P<0.05.

LncFOXO1: long non-coding RNA FOXO1; shRNA: short hairpin RNA; Bax: Bcl-2-associated X; Bcl-2: B cell lymphoma-2

**Figure 5:**
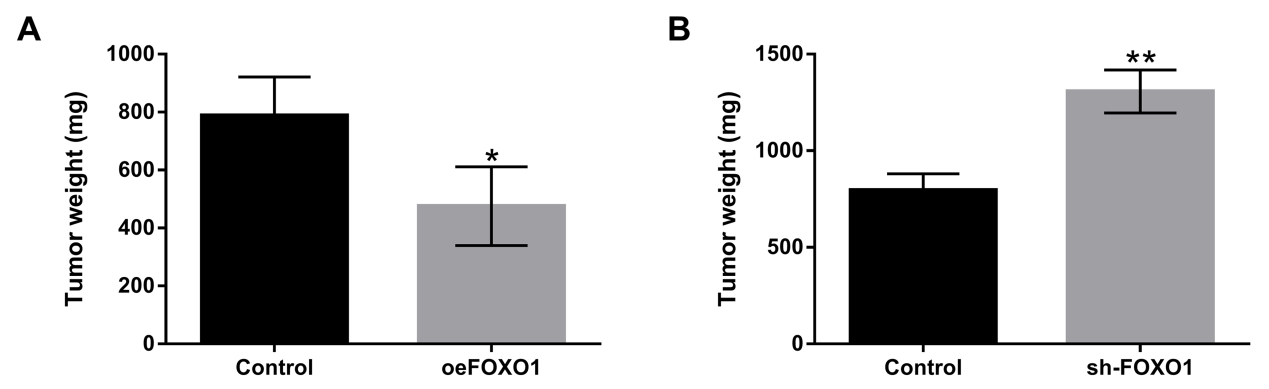
Overexpression of lncFOXO1 inhibited tumor formation in vivo. A549 cells were transfected with pc-FOXO1 and sh-FOXO1 vectors. (A and B) The effect of lncFOXO1 overexpression or lncFOXO1 knockdown on tumor weight was examined by Xenograft tumor model assay. Data are presented as the mean ± SD of three independent experiments; * *P*<0.05, ** *P*<0.01.

LncFOXO1: long non-coding RNA FOXO1; shRNA: short hairpin RNA

**Figure 6:**
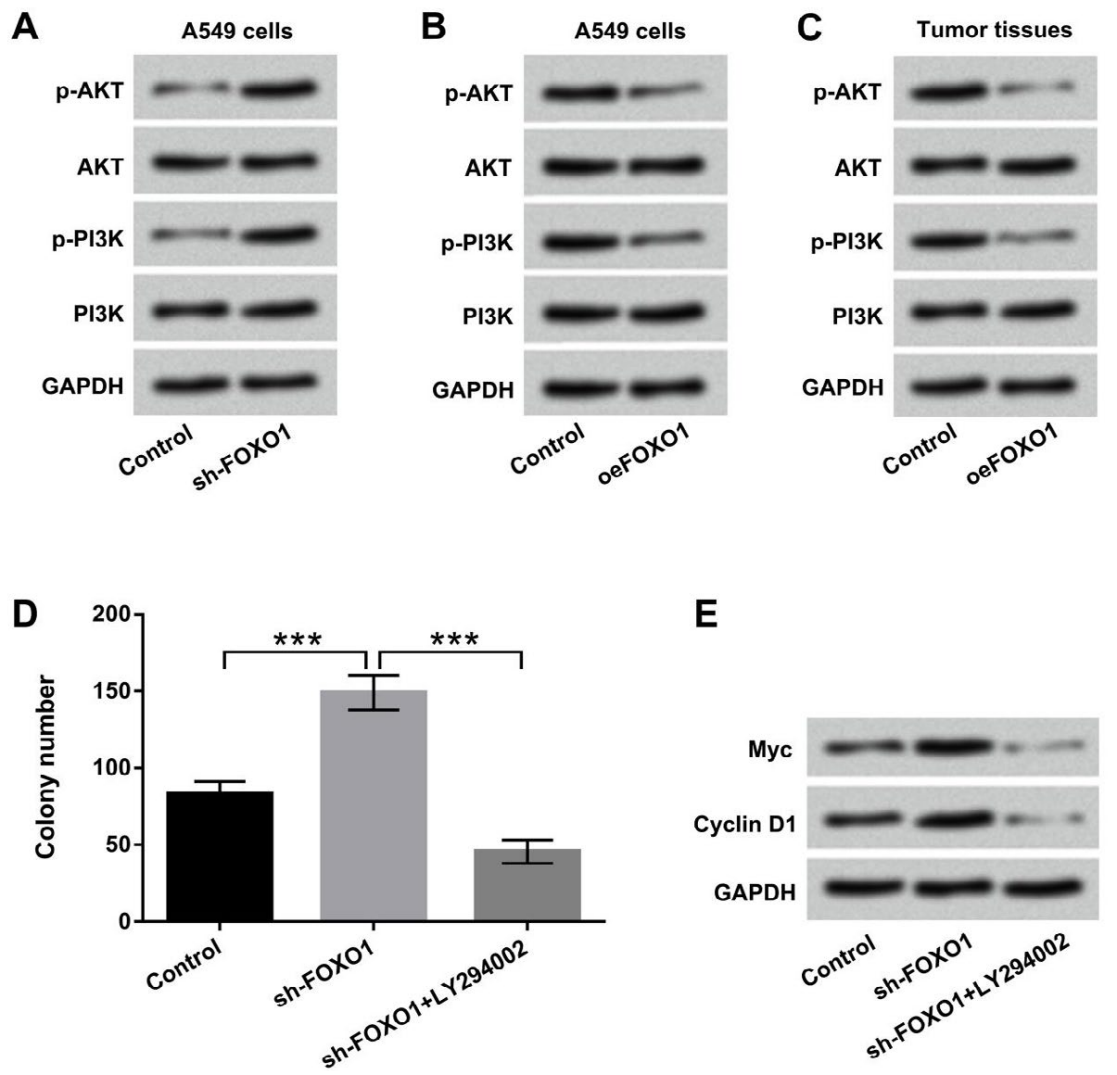
Overexpression of lncFOXO1 exerted anti-proliferative effect by regulating PI3K/AKT signaling pathway. A549 cells were transfected with pc-FOXO1 and sh-FOXO1 vectors. The protein levels of main factors of PI3K/AKT signaling pathway (A) in control and Sh-FOXO1 transfected cells and (B) in control and Oe-FOXO1 transfected cells were analyzed by western blot assay. (C) The protein levels of main factors of PI3K/AKT signaling pathway in tumor tissues was detected by western blot; (D) The colony number in control, sh-FOXO1, sh-FOXO1+LY294002 transfected cells was detected by colony formation assay; (E) The protein levels of Myc and Cyclin D1 in control, sh-FOXO1, and sh-FOXO1+LY294002 transfected cells were measured by western blot. Data are presented as the mean ± SD of three independent experiments; *** *P*<0.001

LncFOXO1: long non-coding RNA FOXO1; shRNA: short hairpin RNA; PI3K: phosphoinositide 3-kinase; AKT: protein kinase B; LY294002: PI3K inhibitor

***Cell culture***

Lung cancer cells (A549, H460, HCC827 and H1299) and lung fibroblast cell line (IMR-90) were obtained from American Type Culture Collection (ATCC, Rockville, MD, USA). These four different lung cancer cell lines were sub-cultured according to the cell instructions. Briefly, cells were transferred to a 75 cm^2^ culture flask from the cell cryopreservation tubes. These cells were cultured in the culture flask containing Roswell Park Memorial Institute (RPMI)-1640 with 10% fetal bovine serum (FBS, Gibco, Carlsbad, CA, USA) and double resistances of penicillin (100 units/ml)-streptomycin (0.1 g/l) (Gibco, Carlsbad, CA, USA) at the condition of 37 ^°^C in an incubator with air (95%) and CO_2 _(5%). IMR-90 used as a control cell line, which was cultured in Dulbecco’s modiﬁed Eagle medium (DMEM, Gibco, Carlsbad, CA, USA) at the same condition as mentioned above.

***Quantitative real time RT-PCR analysis***

Total RNA of A549 cells was extracted by using the common reagent of TRIzol, which was purchased by Invitrogen (Carlsbad, CA, USA). The RNA template (25 μg) was reverse transcribed to cDNA using the PrimeScript RT reagent kit (TaKaRa, Shiga, Japan) according to the manufacturer’s instructions. LncFOXO1 expression was examined by qRT-PCR using the Takara SYBR Premix Ex Taq II (Takara, Shiga, Japan), and was normalized to the GAPDH. LncFOXO1 primer was as follow: Forward, 5’-CGATGTGCTGGAGTGTATGT-3’; Reverse, 5’-GCAGGATGGCACTACTGATAA-3’. GAPDH primer was as follow: Forward, 5’-GATTCCACCCATGGCAAATTC-3’; Reverse, 5’-CTGGAAGATGGTGATGGGATT-3’. All these data were calculated by using the 2^-ΔΔCt ^method (15).

***Cell transfection***

The lncFOXO1 short hairpin RNA (shRNA) was constructed by GenPharma (Shanghai, China) to knockdown lncFOXO1 expression and the full-length lncFOXO1 was cloned into pcDNA3.1 (GenPharma, Shanghai, China) to overexpress lncFOXO1 expression. These cell transfections were conducted by using Lipofectamine 2000 reagent (Invitrogen, Carlsbad, CA, USA) based on the reagent instructions. These cells were collected after transfection for 48 hr, and used for the subsequent experiments.

***Cell viability assay***

The viability of A549 cells was examined by using 3-(4, 5-dimethylthiazol-2-yl)-2 5-diphenyl-2Htetrazolium bromide (MTT) colorimetric assay (Sigma-Aldrich, St. Louis, Mo, USA). Briefly, A549 cells transfected with sh-FOXO1 were collected, and its concentration was adjusted to 5 × 10^3^ cells/well. Then, 200 μl cell suspensions were covered in each well of 96-well plates and incubated for 1, 2, 3 and 4 days under the routine culture condition (37 ^°^C, 5% CO_2_, 95% air). After culturing, 10 μl MTT (5 mg/ml) dye was added into each well of 96-well plates and allowed to complete the reaction for 4 hr. After this, the supernatant was removed, and 100 μl dimethyl sulfoxide (DMSO, Sigma-Aldrich) was added to the each well of culture plates to dissolve the violet crystals. To determine the number of living cells, the absorbance of each well was measured by using a microplate reader (Bio-Rad, Hercules, CA, USA) at a wavelength of 570 nm.

***Colony formation assay***

A549 cells were cultured in 10 cm dishes and maintained at 37 ^°^C in 5% CO_2_, and the culture medium was replaced every 3-4 days. After three weeks, A549 cells were fixed in 100% methanol. After this, 400 μl crystal violet (0.1%, Merck, Darmstadt, Germany) was added to the culture dishes to stain the cells for 15-30 min. The colony formation cells were finally counted under a microscope (Bio-Rad, Hercules, CA, USA). Three replicate wells were included for each group. 

***Ki67 positive cells analysis***

After transfection with oeFOXO1 for 48 hr, transfected A549 cells were collected and washed with phosphate-buffered saline (PBS). Then, the transfected cells were fixed in chilled methanol-acetone for 10 min at -20°C. Afterward, these cells were incubated with 3% bovine serum albumin (BSA, Roche, Indianapolis, IN, USA) for 1 hr, and were then incubated with an anti-Ki67 antibody (ab15580, dilution of 1:100, Abcam, Cambridge, UK) for another 1 hr at 4 ^°^C. After the above treatments were completed, the sterile PBS was used to wash these cells for three times, and cells were then incubated with an anti-rabbit antibody (ab205718, Abcam, Cambridge, UK) conjugated with FITC for 4 hr at 37 ^°^C. FACScan flow cytometry assay (Beckman Coulter, Fullerton, CA, USA) was performed to analyze the percentage of the positive stained cells in this study. 

***Cell invasion and migration assays***

After transfection with sh-FOXO1 and oeFOXO1, the viabilities, invasion and migration of A549 cell were analyzed as described previously (16). For cell invasion assay, 24-well plate Transwell chamber covered with BD MatrigelTM Matrix (BD Biosciences, NY, USA) was used. Briefly, cells (5 × 10^4^) were re-suspended in 200 μl serum-free medium and were incubated in the upper chamber. Then, 750 μl complete medium was added to the basolateral chamber. After incubation for 24 hr at 37 ^°^C, the culture medium in the Transwell chamber was wiped off, and the non-invasive cells were removed with a wet cotton swab gently. Subsequently, these cells were fixed and stained with 0.1% crystal violet (Merck, Darmstadt, Germany) for 30 min. The stained cells were then counted using a microscope (Leica Microsystems, Wetzlar, Germany). The migration assay was performed with the same steps as the invasion assay, but the membrane was not coated with matrigel (BD Bioscience, NY, USA).

***Apoptosis assay***

Cell apoptosis of A549 cells transfected with oeFOXO1 was determined by using Annexin V-Phycoerythrin (PE)/7-aminoactinomycin D (7-ADD) apoptosis detection kit (Beijing Biosea Biotechnology, Beijing, China). In brief, the cells (1 × 10^6^ cells/well) were cultured in 6 well-plates under the regular culture conditions. After transfection with oeFOXO1, A549 cells were washed three times by using pre-cold PBS (Sigma-Aldrich). Subsequently, these cells were re-suspend in 500 μl 1 × binding buffer and 5 μl Annexin V-PE (Beijing Biosea Biotechnology), and 5 μl 7-AAD (Beijing Biosea Biotechnology) was added to the cell suspensions and incubated for 15 min at room temperature in the dark. The percentage of apoptotic cells was finally measured by using flow cytometry (Beckman Coulter, Fullerton, CA, USA) assay.

***Tumor growth in nude mice***

The female nude BALB/c mice (specific pathogen-free grade, five-week old) were obtained from Vital River Laboratory (VRL, Beijing, China). To explore the suppressive effects of LncFOXO1 *in vivo*, the A549 cells transfected with oeFOXO1 and sh-FOXO1 were harvested and washed in PBS for 2 times. Afterward, 5 × 10^6^ cells were inoculated subcutaneously into fossa axillaris of these nude mice. After five weeks, the tumor weights were collected and measured. All experiments associated with mice were approved by The Affiliated Hospital of Qingdao University.

***Western blot assay***

Western blot assay was carried out to examine the protein levels of matrix metallopeptidase 9 (MMP9), MMP2, Bax, cleaved-caspase-3, Bcl-2 and PI3K/AKT signal pathway in A549 cells transfected with oeFOXO1, sh-FOXO1 or sh-FOXO1 together with LY294002 (PI3K/AKT inhibitor). Briefly, the protein sample was extracted by using radio immunoprecipitation assay (RIPA) lysis buffer (Beyotime Biotechnology, Shanghai, China). The BCA Protein Assay Kit (Pierce, Rockford, IL, USA) was used to quantify the concentration of total protein. Then, 30 μg of cell lysates were subjected to sodium dodecyl sulfate-polyacrylamide gel electrophoresis (SDS-PAGE), and then transferred to polyvinylidenedifluoride (PVDF) membranes. After blocking with 5% BSA, these membranes were incubated with specific antibodies for MMP9 (ab38898, 1:1000), MMP2 (ab92536, 1:1000), Bax (ab32503, 1:1000), cleaved-caspase-3 (ab32042, 1:500), Bcl-2 (ab32124, 1:1000), phosphorylated (p)-AKT (ab38449, 1:500), AKT (ab8805, 1:500), p-PI3K (ab182651, 1:500), PI3K (ab180967, 1:2000), Myc (ab39688, 1:500), Cyclin D1 (ab16663, 1:200) and GAPDH (ab181603, 1:10000, Abcam, Cambridge, UK) at 4 °C overnight. The membranes were then washed with Tris-buffered saline and Tween 20 (TBST) for several times, and incubated with the secondary antibody of horseradish peroxidase (HRP)-conjugated goat anti-rabbit IgG (ab205718, 1:2000, Abcam) for another 1 hr at room temperature. The signals were exposed by ECL reagents (MultiSciences Biotech, Hangzhou, China) according to the reagent instructions.

***Statistical analysis***

Data from the current study are presented as the mean±standard deviation (SD), and the statistical analyses were carried out by using SPSS 19.0 statistical software (SPSS, Inc., Chicago, IL, USA). Student’s *t*-test and a one-way analysis of variance (ANOVA) followed by Duncan *post-hoc *test were used to analyze the significant differences of two groups or multiple groups. Significances were indicated at *P*<0.05.

## Results

***LncFOXO1 was down-regulated in lung cancer tumor tissues and cells***

The study aimed to uncover the effect of lncFOXO1 on lung cancer, so lncFOXO1 expression level was examined in lung cancer tumor tissues, firstly. In Figure 1A, the results showed the significant decrease of lncFOXO1 expression in lung cancer tumor tissues compared to that in adjacent tissues (*P*<0.001). Further, we selected four lung cancer cell lines (A549, H460, HCC827 and H1299) to explore the effect of lncFOXO1 on lung cancer cells. As shown in Figure 1B, the expression of lncFOXO1 in A549, H460, HCC827 and H1299 cells were significantly declined compared to that in IMR-90 cells (*P*<0.05, *P*<0.01 or *P*<0.001). These results suggested that lncFOXO1 was down-regulated in lung cancer tumor tissues and cells. A549 cell line was selected for the following experiments.

***Knockdown of lncFOXO1 promoted cell proliferation and invasion***

Above results showed an abnormal expression of FOXO1 in lung cancer tissues and cells. We suspected that FOXO1 might be involved in regulating the biological processes of lung cancer cells. To confirm the supposition, we investigated A549 cells proliferation and invasion after transfection with sh-FOXO1 vector. The results showed that the expression level of lncFOXO1 was significantly decreased in sh-FOXO1-transfected A549 cells compared to control group (*P* <0.001, Figure 2A). The viability of A549 cells was promoted by knockdown of lncFOXO1 at day three and day four compared to control group (*P*<0.05, Figure 2B). The colony number was also increased by knockdown of lncFOXO1 (*P*<0.01, Figure 2C). Furthermore, we examined the invasive ability of A549 cells by using Transwell assay. The results displayed that lncFOXO1 knockdown remarkably enhanced the invasiveness of A549 cells compared to control group (*P*<0.05, Figure 2D). These data together uncovered the promoting effect of lncFOXO1 knockdown on cell proliferation and invasion in A549 cells.

***Over-expression of lncFOXO1 suppressed cell proliferation and migration***

We used overexpress vector to alter lncFOXO1 expression in A549 cells to explore the functions of lncFOXO1 in lung cancer cells. In Figure 3A, we observed that the expression level of lncFOXO1 was significantly up-regulated in oeFOXO1-transfected A549 cells compared to that in control group (*P*<0.001). Cell viability was significantly reduced by overexpression of lncFOXO1 compared to control group at day three and day four (*P*<0.05 or *P*<0.01, Figure 3B). Subsequently, we found that lncFOXO1 overexpression significantly declined the percentage of Ki67^+^ cells compared to control group (*P*<0.05, Figure 3C). The migration ability of A549 cells was also reduced by lncFOXO1 overexpression compared to control group (*P*<0.05, Figure 3D). Furthermore, western blot results revealed that lncFOXO1 overexpression obviously down-regulated MMP9 and MMP2 expression levels compared to control group (Figure 3E). In sum, these data indicated the inhibitory effect of lncFOXO1 overexpression on cell proliferation and migration in A549 cells.

***Overexpression of lncFOXO1 induced cell apoptosis in A549 cells***

Cell apoptosis was examined by flow cytometry in A549 cells transfected with oeFOXO1 vector. As shown in Figure 4A, the results revealed that lncFOXO1 overexpression significantly induced the percentage of apoptotic cells compared to control group (*P*<0.05). Western blot assay results showed that overexpression of lncFOXO1 up-regulated Bax and cleaved -caspase-3 expressions, and down-regulated Bcl-2 expression in A549 cells (Figure 4B). However, knockdown of lncFOXO1 showed opposite results in Bax, cleaved -caspase-3 and Bcl-2 expression in A549 cells (Figure 4C). According to all above, these data indicated the promoting effect of lncFOXO1 overexpression on cell apoptosis in A549 cells.

***Overexpression of lncFOXO1 inhibited tumor formation in vivo***

To further verify the suppressive effect of lncFOXO1 on lung cancer, the overexpression and knockdown vectors of lncFOXO1 were transfected into A549 cells, and then tumor weight was examined by Xenograft tumor growth assay* in vivo*. The results displayed that overexpression of lncFOXO1 significantly declined tumor weight, but knockdown of lncFOXO1 enhanced tumor weight compared to control group (*P*<0.05 or *P*<0.01, Figure 5A and 5B). These data explained the inhibitory effect of lncFOXO1 overexpression on tumor formation *in vivo*.

***Overexpression of lncFOXO1 exerted anti-proliferative effect by regulating PI3K/AKT signaling pathway***

To explore the mechanism of the suppressive effect of lncFOXO1 on lung cancer, PI3K/AKT signaling pathway was explored. Results displayed that knockdown of lncFOXO1 up-regulated the expression of phosphorylated AKT and PI3K in A549 cells (Figure 6A). However, overexpression of lncFOXO1 notably down-regulated phosphorylated AKT and PI3K expression in A549 cells (Figure 6B). LncFOXO1 overexpression also decreased the expressions of these two factors in tumor tissues (Figure 6C). Additionally, LY294002 was added to cells to inhibit PI3K/AKT signaling pathway. As shown in Figure 6D, knockdown of lncFOXO1 together with LY294002 significantly decreased colony number compared to knockdown of lncFOXO1 group (*P* < 0.001). Besides, the protein levels of Myc and Cyclin D1 were decreased by knockdown of lncFOXO1 together with LY294002 compared to knockdown of lncFOXO1 group (Figure 6E). Taken together, these data indicated that lncFOXO1 overexpression exerted anti-proliferative effect might be through regulating PI3K/AKT signaling pathway.

## Discussion

In the present study, we performed *in vitro* and *in vivo *experiments to uncover the effect of lncFOXO1 on lung cancer. Down-regulation of lncFOXO1 was found in lung cancer tissues and cells. Moreover, overexpression of lncFOXO1 obviously inhibited cell proliferation, migration, invasion and promoted apoptosis in A549 cells. *In vivo* experiment demonstrated that lncFOXO1 overexpression inhibited tumor formation. Besides, lncFOXO1 overexpression exhibited anti-proliferative effect by regulating PI3K/AKT signaling pathway.

LncRNA, a by-product of RNA polymerase II transcription, is originally thought to be the “noise” of genome transcription without biological function (17). However, recent studies showed that lncRNA was closely associated with the processes of X-chromosome silencing, genomic imprinting, chromatin modification, and transcriptional activation (18). Moreover, increasing evidences demonstrated that lncRNAs play vital roles in the processes of cell proliferation, differentiation, metastasis and apoptosis in different cancers, including lung cancer (19-21). For instance, Nie *et al.* revealed the promoting effect of lncRNA antisense non-coding RNA in the INK4 locus (ANRIL) on cell proliferation and the inhibitory effect of ANRIL on apoptosis in lung cancer cells, and the functions might be achieved through silencing of Kruppel-like factor 2 (KLF2) and P21 expression (22). *In vitro* experiment from Zhao *et al.* uncovered the effect of lncRNA HOX transcript antisense intergenic RNA (HOTAIR) on lung cancer cell motility and invasion (23). LncFOXO1 is recently discovered as a novel lncRNA, which is declined in breast cancer tissues and cells; in addition, lncFOXO1 has been proven to play a suppressive role in breast cancer (14). 

Based on these studies, increasing interest has been brought to bear on exploring the effect of lncFOXO1 on lung cancer cells. Our study showed that lncFOXO1 was declined in lung cancer tissues and cells, and overexpression of lncFOXO1 suppressed cell proliferation, invasion and migration in A549 cells. Moreover, our study demonstrated that overexpression of lncFOXO1 promoted cell apoptosis by regulating pro-apoptotic factor (Bax), anti-apoptotic factor (Bcl-2) and cleaved-caspase-3 expression in A549 cells. Furthermore, to clarify the tumor suppressing effect of lncFOXO1 on lung cancer, we carried out the Xenograft tumor model assay to measure tumor weight *in vivo*. We found that lncFOXO1 overexpression significantly inhibited tumor weight. These data further indicated the suppressive effect of lncFOXO1 on lung cancer. 

Accumulating evidences have demonstrated that PI3K/AKT signaling pathway acts as key regulator in the development of different cancers (24-26). Activation of PI3K/AKT signaling pathway has been clarified to be associated with various biological processes, such as cell proliferation, apoptosis, invasion and migration (27, 28). Recent study of Wang *et al.* revealed that lncRNA AB073614 knockdown exerted anti-proliferative effect on colorectal cancer cells by regulating PI3K/AKT signaling pathway (29). Liu *et al.* found that up-regulated lncRNA colorectal neoplasia differentially expressed (CRNDE) could increase lung cancer cells proliferation by mediation of PI3K/AKT signaling pathway (30). However, whether lncFOXO1 exerted suppressive effect via mediating PI3K/AKT signaling pathway remains unclear. Our results revealed that lncFOXO1 overexpression significantly declined the activation of PI3K/AKT signaling pathway in A549 cells and lung cancer tumor tissues. Further, knockdown of lncFOXO1 together with LY294002 significantly decreased colony number and Myc and Cyclin D1 expressions. These data indicated that lncFOXO1 overexpression exerted anti-proliferative effect by inactivation of PI3K/AKT signaling pathway.

## Conclusion

Collectively, the results of the present study demonstrated that lncFOXO1 could inhibit lung cancer cell proliferation, migration, and invasion, and promote apoptosis via down-regulating PI3K/AKT signaling pathway. The study firstly demonstrated the tumor suppressive effect of lncFOXO1 on lung cancer, and these finding might provide a helpful strategy for the treatment of lung cancer in the clinic. LncFOXO1 might become a therapeutic target of lung cancer patients in the future. Further studies are still necessary to clarify these hypotheses.
